# Public health round-up

**DOI:** 10.2471/BLT.16.010416

**Published:** 2016-04-01

**Emergencies in more than 30 countries**

This mother and child in Ethiopia’s Somali region are among the eight million people worldwide coping with the effects of El Niño and needing food assistance. The World Health Organization (WHO) is responding to emergencies in more than 30 countries. Last month, WHO joined partners at a conference in Lausanne, Switzerland to promote innovative solutions to humanitarian challenges as part of the Global Partnerships for Humanitarian Impact and Health initiative of the International Committee of the Red Cross. http://blogs.icrc.org/gphi2/about-gphi2/

**Figure:**
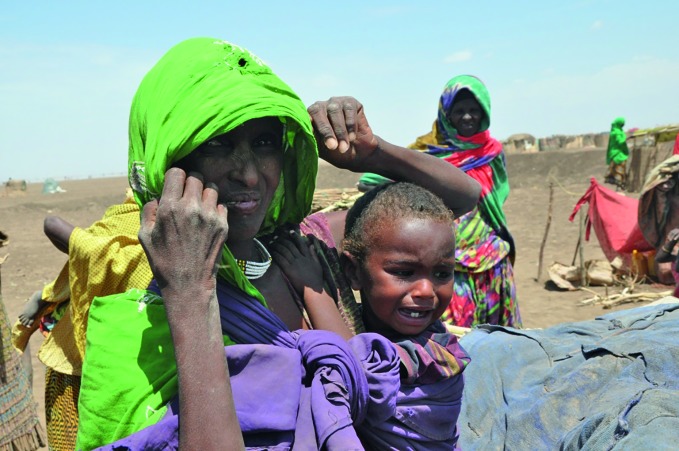
WHO/Kibnesh Chala

## Protecting pregnant women 

People living in areas affected by Zika virus should take measures to protect themselves from mosquito-bites and destroy mosquito habitats to prevent pregnant women becoming infected with Zika virus, according to WHO interim guidance issued last month. 

Zika virus infection in pregnancy is typically a mild disease, but an unusual increase in cases of neurological disorders and neonatal malformations in areas where Zika outbreaks have occurred, has raised concern for pregnant women and their families.

WHO issued interim guidance on steps that national and local health managers can take when developing protocols and health policies related to pregnancy care in the context of Zika virus transmission.

“The importance of preventive measures should be emphasized [by health professionals] at every contact with a pregnant woman,” the guidance says. 

These preventive measures include mosquito bednets, including when sleeping during the daytime; mosquito mesh/nets/screens on windows and doors; insect repellents and clothes that cover as much of the body as possible. 

To prevent potential sexual acquisition of Zika virus during pregnancy, the sexual partners of pregnant women, living in or returning from areas of ongoing Zika virus transmission, should, for the duration of the pregnancy, use condoms or adhere to other safe sexual practices, including abstinence, the guidance says.

In addition, safe sex practices are recommended for people infected with Zika, dengue and chikungunya virus, including consistent and correct use of condoms, for at least four weeks after onset of symptoms to avoid further spread of these infections, the guidance says.

The guidance also provides advice on the diagnosis and care of pregnant women with Zika infection. 

http://www.who.int/csr/resources/publications/zika/pregnancy-management/

## Psychosocial guidance and Zika

Health professionals should take a supportive approach when they diagnose women with Zika virus or attend to the families of babies with neonatal malformations, such as microcephaly, and neurological disorders in areas affected by Zika virus, according to interim WHO guidance.

The guidance is for health professionals attending to three groups: pregnant women with suspected or confirmed Zika virus infection; pregnant women who know they are carrying a child with suspected microcephaly; and the caregivers and families of an infant with microcephaly. 

“The way a health-care provider assesses and manages these health conditions can have an impact on the psychosocial well-being of patients and their families”, the guidance says, stressing the importance of providing reliable information in a form that women and their partners understand and of asking them about their concerns. 

This practical guidance also offers advice on stress management, strengthening social supports and parenting.

http://www.who.int/csr/resources/publications/zika/psychosocial-support/

## Zika research 

WHO held a three-day consultation last month on the status of Zika clinical research, looking at ways to drive research and development on vaccines, medicines, vector control and diagnostics needed to respond to the Zika crisis. 

Scientists and health decision-makers – including those from Zika-affected countries – as well as regulators and industry representatives took part in the 7–9 March meeting in Geneva. 

The Geneva gathering built on a meeting a week earlier in Washington DC, the United States of America, at the WHO Regional Office for the Americas and a visit by Regional Director Dr Carissa F Etienne and WHO Director-General Dr Margaret Chan to Brazil. 

In Brazil, Chan and Etienne met President Dilma Rousseff and other top Brazilian officials to assess the Zika virus situation and response. 

The two WHO officials visited Brazil’s National Center for Risk and Disaster Management (CENAD) for discussions with government ministers and also visited the Professor Fernando Figueira Integral Medicine Institute, the national referral centre for mother and child care programmes in Recife, Pernambuco.

Pernambuco is the Brazilian state where a significant number of pregnant women who contracted Zika virus during pregnancy delivered babies with microcephaly last year.

http://www.who.int/features/2016/zika-brazil/

**Cover photo**

Students enjoy a warm meal in their classroom at Kanda Estate Primary School in Accra, Ghana.

**Figure:**
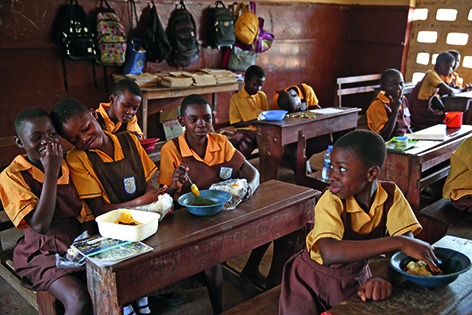
World Bank/Dominic Chavez

## Health worker commission starts work

A High-Level Commission on Health Employment and Economic Growth, appointed by United Nations Secretary-General Ban Ki-moon met in Lyon, France last month to discuss ways to address the global mismatch in the supply, demand and need for health workers.

Low- and lower-middle-income countries need an additional 18 million health workers if they are to make progress towards providing universal coverage of health services and achieve the sustainable development goals’ health targets by 2030.

At the same time, demand for health workers is projected to create about 40 million new jobs in health care, primarily in upper-middle and high-income countries, which may encourage international migration of health workers. 

The new commission is chaired jointly by President Francois Hollande of France and President Jacob Zuma of South Africa. WHO, the International Labour Organization and the Organisation for Economic Co-operation and Development are acting as co-vice chairs.

It is due to deliver its recommendations to the Secretary-General during the 71st session of the United Nations General Assembly in May. 

The General Assembly asked the Secretary-General last December to look into ways of meeting the global shortfall of trained health workers, within the context of achieving universal health coverage and the sustainable development goals. 

http://www.who.int/hrh/com-heeg/

## New Hepatitis C recommendations

Patients with hepatitis C virus infection should be treated – whenever possible – with direct-acting antivirals rather than interferon-containing regimens that were previously recommended, according to updated WHO recommendations published last month. 

This is one of several recommendations in *Guidelines for the screening, care and treatment of persons with hepatitis C infection.*

Since the document was first issued in 2014, many new drugs have been licensed for hepatitis C virus infection and some of them were included in the latest *WHO Model list of essential medicines* released last year. 

The updated guidelines for the treatment of hepatitis C virus infection reflect these changes and include new evidence-based recommendations. 

“These medicines are transforming the treatment of hepatitis C virus infection, enabling the use of regimens that can be administered orally, [that] are shorter in duration (as short as eight weeks), [and that] result in cure rates higher than 90%, and are associated with fewer serious adverse events,” according to the updated guidelines. 

The updated guidelines no longer recommend treatment with first-generation protease inhibitors, telaprevir and boceprevir, as these two medicines are less effective than newer medicines and have more frequent adverse effects. 

The updated guidelines also offer recommendations on the preferred and alternative regimens based on a patient’s hepatitis C virus genotype and clinical history, such as whether patients have cirrhosis or not. 

About 500 000 persons die every year as a result of hepatitis C virus-related complications, including hepatocellular carcinoma and cirrhosis of the liver. 

Hepatitis C virus infection can be cured by antiviral treatment, but the new drugs are unaffordable in many countries. Fortunately, prices are declining in some countries through the introduction of generics and negotiated price reductions with manufacturers. 

Despite the fact that prices are falling, “the scaling up of treatment is likely to remain restricted in many countries because of the continued high prices of medicines and lack of health-care infrastructure,” according to the updated guidelines. 

Hepatitis C is a bloodborne virus most commonly transmitted through the use of shared or unsterilized injection equipment and through unscreened blood transfusions. 

http://www.who.int/hepatitis/publications/hepatitis-c-guidelines-policy/

**Looking ahead**
24–30 April – World Immunization Week.
25 April – World Malaria Day.
23–28 May – Sixty-ninth World Health Assembly, Geneva, Switzerland.
31 May – World No Tobacco Day.
14 June – World Blood Donor Day.

